# Oxidative modulations in platelets stored in SSP+, PAS-G and Tyrode's buffer: a comparative analysis

**DOI:** 10.1016/j.htct.2024.04.121

**Published:** 2024-08-18

**Authors:** Magdaline Christina Rajanand, Anusha Berikai Ananthakrishna, Vani Rajashekaraiah

**Affiliations:** School of Sciences, JAIN (Deemed-to-be University), Bengaluru, India

**Keywords:** Blood banking, Blood platelets, Blood transfusion, Oxidative stress, Reactive oxygen species

## Abstract

**Background:**

Platelet additive solutions (PASs) improve the efficacy of stored platelets. Oxidative stress causes storage lesions and platelet functions deteriorate. Studies assessing the influence of oxidative stress on platelets stored in PASs are limited. This study compares variations in platelets in different storage solutions (SSP+, PAS-G and Tyrode's buffer).

**Methods:**

Platelets isolated from the blood of Wistar rats were resuspended in SSP+, PAS-G and Tyrode's buffer and stored for seven days at 22 °C. The markers of platelet metabolism, function, oxidative stress, antioxidant status and viability were analyzed on Days 1, 3, 5 and 7 of storage.

**Main results:**

SSP+ is associated with platelet function, viability and antioxidant defenses (SOD, CAT and GSH); it decreased primary lipid peroxidation products and maintained the susceptible protein groups in reduced state. Platelet function, antioxidant defenses such as SOD and GSH improved, and lipids and thiols were protected from oxidation in PAS-G. SOD and GSH increased, and lipids and thiols were preserved in Tyrode's buffer.

**Conclusion:**

SSP+ and PAS-G are more effective in maintaining platelet efficacy till Day 7 compared to Tyrode's buffer. Thus, PAS-G and SSP+ are better than Tyrode's buffer in terms of platelet responses to oxidative stress during storage. This is the first comparative account on the influence of PASs (SSP+, PAS-G and Tyrode's buffer) on platelets in altering oxidative stress. It provides a comprehensive view of the differential responses of platelets in PASs.

## Introduction

The shelf-life of platelets during storage in plasma at 22–24 °C is limited to 3–5 days as they are prone to storage lesions and bacterial contamination beyond this period. Platelet additive solutions (PASs), as a substitute for plasma, reduce the risk of allergic reactions and transfusion-related acute lung injury (TRALI) post-transfusion.^1^ PASs have proven to improve the efficacy of stored platelets and extend their shelf-life to up to nine days. Salts present in PASs provide the buffering capacity and maintain the quality of stored platelets. SSP+ contains 30 % plasma in addition to salts whereas, PAS-G contains glucose.^2^ Tyrode's buffer preserves cells in optimal conditions and thereby, the risk of contamination is reduced during storage.^3^ SSP+ and PAS-G provide enhanced buffering capacity when compared to Tyrode's buffer as they contain citrate, acetate and magnesium.

Platelets are metabolically active resulting in release of reactive oxygen species (ROS), causing oxidative stress (OS), which leads to diminished platelet quality during storage.^4^

There are few OS studies concerning SSP+^5^^,^^6^ and Tyrode's buffer^3^ whereas, the evaluation of metabolic and functional markers have been reported for PAS-G.^7^ The mechanisms leading to storage lesion in terms of OS are still unclear. Therefore, there is a necessity to understand the intermittent changes and response of platelets in PASs. Hence, this study aims to investigate the variations of OS and compare the response of platelets in three different storage solutions (SSP+, PAS-G and Tyrode's buffer).

## Materials and methods

This study was conducted in accordance with the regulations of institutional ethical committee (841/b/04/CPCSEA).

### Blood sampling

Male Wistar rats (four months old) were anesthetized and restrained in dorsal recumbency. Blood was carefully aspirated from the heart into collection tubes with citrate phosphate dextrose adenine (CPDA-1).^3^

### Isolation of platelets

Whole blood was centrifuged at 1500 rpm for 15 min at room temperature. The platelet-rich-plasma obtained was then centrifuged at 4000 rpm for 15 min at 22 °C.^3^ The resulting platelet pellet was gently resuspended in platelet storage solutions – SSP+ (pH 7.4), PAS-G (pH 7.0) and Tyrode's buffer (Tyrode's) (pH 7.4).

### Experimental design

Platelets were divided into three groups (the blood of five rats was used for each group) and were resuspended in SSP+, PAS-G and Tyrode's and stored in polypropylene tubes at 22 °C for a period of seven days. Markers of platelet metabolism, function, OS and antioxidant defenses were assayed on Days 1, 3, 5 and 7 of storage.

### Platelet metabolism

#### pH

The pH in the platelet samples was determined using Fisher Scientific pH strips.^8^

### Glucose

Glucose in the platelets was measured by the glucose oxidase-peroxidase (GOD-POD) method by following the protocol described for the Autospan Gold kit. Light absorbance was measured at 546 nm.^9^

#### Lactate dehydrogenase (LDH, EC 1.1.1.27)

Platelets were treated with the mixture of Reagent 1 (Tris, NaOH and pyruvate) and Reagent 2 (Nicotinamide adenine dinucleotide) and incubated at 37 °C. Light absorbance was measured at 340 nm.^10^

### Platelet function

#### Platelet aggregation

Platelets were incubated with and without collagen (2.0 µg/mL) at 37 °C. Light absorbance was measured at 405 nm.^11^

#### Adenosine triphosphate (ATP) secretion

Platelets were incubated at 37 °C with collagen (2.0 µg/mL) and treated with 1.2 M perchloric acid. Light absorbance was read at 260 nm and the amount of adenine nucleotides was calculated using an ATP standard.^12^

### Oxidative stress

#### Superoxides

Platelets were incubated with 200 µL cytochrome C (160 µM) at 37 °C. The cytochrome C reduction was measured spectrophotometrically at 550 nm.^13^

#### Nitrites

Platelets were treated with Griess reagent and incubated in the dark at room temperature. Light absorbance was measured at 548 nm. Sodium nitrite was used as the standard to determine the amount of nitrites.^14^

### Lipid peroxidation

#### Conjugated dienes

Platelets were treated with ether/ethanol (1:3 (v/v)). The mixture was centrifuged and light absorbance was measured spectrophotometrically at 235 nm.^13^

#### Thiobarbituric Acid Reactive Substances (TBARS)

Platelets treated with 20 % cold trichloroacetic acid in 0.6 M HCl were centrifuged and 0.12 M thiobarbituric acid was added to the supernatant. The samples were incubated in a boiling water bath and light absorbance was read at 532 nm.^15^

### Protein oxidation

#### Protein sulfhydryls

Platelets were treated with a sodium phosphate buffer (0.08 M) containing sodium ethylenediaminetetraacetic acid (Na_2_-EDTA - 0.5 mg/mL) and sodium dodecyl sulphate (2 %). 5,5′-dithiobis-2-nitrobenzoic acid (DTNB) was added and the color developed was measured at 412 nm with the amount of sulfhydryls being calculated using the molar absorptivity of 13,600/M/cm.^16^

#### Advanced oxidation protein products (AOPP)

Platelets were treated with isotonic phosphate buffer, 1.16 M potassium iodide, and glacial acetic acid. Light absorbance was measured at 340 nm. The amount of AOPP was estimated using an extinction coefficient of 26/mM/cm.^17^

### Antioxidant status

#### Superoxide dismutase (SOD, EC 1.15.1.1)

Carbonate buffer (0.05 M; pH 10.2; 0.1 mM EDTA) was added to platelets, followed by epinephrine with light absorbance being measured at 480 nm. The SOD activity was expressed as the amount of enzyme that inhibits oxidation of epinephrine by 50 %, which is equal to 1 unit.^18^

*Catalase (CAT, EC 1.11.1.6)*

Platelets were treated with absolute ethanol and incubated in an ice bath (30 min). After incubation, phosphate buffer and 0.066 M hydrogen peroxide were added and light absorbance was measured at 240 nm. The catalase activity was determined using the molar extinction coefficient 43.6/M/cm.^19^

### Glutathione

Platelets were treated with 4 % sulfosalicylic acid, vortexed, and centrifuged at 2500 g for 15 min. The supernatant was treated with 10 mM DTNB and light absorbance was read at 412 nm.^20^

*Total Antioxidant Capacity_Cupric ion reducing antioxidant capacity_ (TAC_CUPRAC_)*

Platelets were treated with bathocuproinedisulfonic acid disodium salt (0.25 mM) and light absorbance was measured at 490 nm. The CuSO_4_ solution (0.5 mM) and EDTA solution (0.01 M) were added and the final light absorbance was measured. Uric acid was used as the standard.^21^

### Platelet quality

#### Cell viability

Platelets along with 3-(4,5-dimethylthiazol-2-yl)−2,5-diphenyl tetrazolium bromide reagent (0.5 mg/mL) were incubated for 4 h (37 °C). Dimethyl sulfoxide was added and incubated overnight.^22^ Light absorbance was measured at 493 nm.

#### Protein

Protein concentration in platelets were determined according to Lowry et al.

### Statistical analyses

The results are represented as means ± standard error (SE) (*n* = 5). Two-way analysis of variance (ANOVA) was performed between the groups (storage days) and sub-groups (between the storage solutions). The results were considered significant at *p*-values < 0.05. The Bonferroni *post hoc* test was applied using GraphPad Prism-8 Software.

## Results

i) First paragraph: Comparison between the storage days with Day 1 of each storage solution.
ii) Second paragraph: Comparison of platelet response in SSP+, PAS-G and Tyrode's on each day of storage.

### Metabolic markers

#### pH

The pH increased by 20 % (*p*-value <0.05) on Days 5 and 7 in SSP+ with respect to Day 1 whereas, pH was maintained throughout the storage period in PAS-G and Tyrode's.

**Figure 1:**
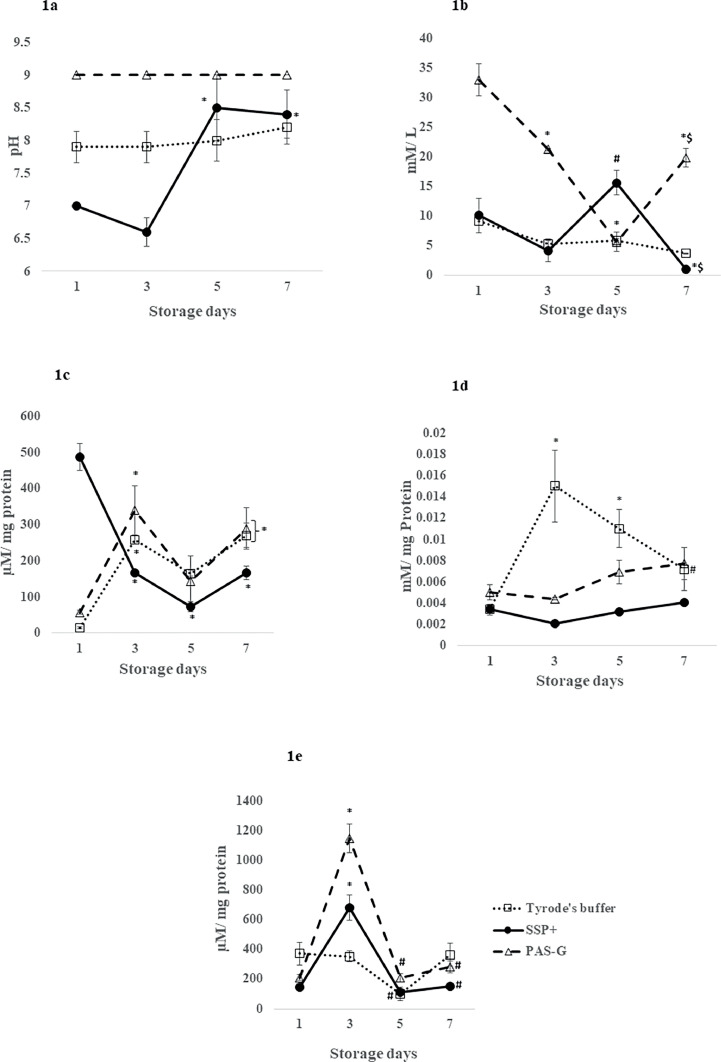
Metabolism (pH, glucose), protein oxidation (sulfhydryls, AOPP) and lipid peroxidation (conjugated dienes) in different storage solutions. Values are means ± standard error (SE) of the platelets of five animals per group. Two-way ANOVA was applied between the groups (storage days) and sub groups (storage solutions) followed by Bonferroni's *post hoc* test using Graph Pad Prism-8 software. *P*-values <0.05 were considered significant. *Represents significant changes between the groups with respect to Day 1. ^#^Represents significant changes between the groups with respect to Day 3. ^$^Represents significant changes between the groups with respect to Day 5. 1a. pH: pH incremented in PAS-G (*p*-value <0.0001) and Tyrode's buffer (*p*-value <0.05) compared to SSP+ on Days 1 and 3. 1b. Glucose: Glucose increased in PAS-G (*p*-value <0.0001) on Day 1 however, it peaked in SSP+ (*p*-value <0.01) on Day 5 compared to other storage solutions. 1c. Protein sulfhydryls: Sulfhydryls increased in SSP+ compared to PAS-G and Tyrode's buffer on Day 1 (*p*-value <0.0001). 1d Advanced Oxidation Protein Products (AOPP): SSP+ and PAS-G exhibited increments in AOPP compared to Tyrode's buffer on Days 3 (*p*-value <0.0001) and 5 (*p*-value <0.01). 1e. Conjugated dienes: Conjugated dienes increased in PAS-G compared to SSP+ and Tyrode's buffer (*p*-value <0.0001) on Day 3 (*p*-value <0.0001).

The pH increased in Tyrode's (15 %) (*p*-value <0.05) on Day 3 and in PAS-G on Days 1 (28 %) and 3 (36 %) (*p*-value <0.0001) compared to SSP+ (Figure 1a).

#### Glucose

Glucose peaked by 200 % (*p*-value <0.001) on Day 5 with respect to Day 3 and decreased by 90 % (*p*-value <0.05) on Day 7 in SSP+ with respect to Day 1. Glucose showed a gradual decreases of 35 % and 83 % (*p*-value <0.001) on Days 3 and 5. and increased (*p*-value <0.0001) on Day 7 to Day 3 levels in PAS-G. Glucose was maintained throughout storage in Tyrode's.

PAS-G showed increments (*p*-value <0.0001) of 2-, 3- and 4-fold with Tyrode's and 2-, 4- and 10-fold with SSP+ on Days 1, 3 and 7. However, glucose elevated by 1-fold (*p*-value <0.01) on Day 5 in SSP+ compared to the other storage solutions (Figure 1b).

#### Lactate dehydrogenase (LDH)

LDH showed similar variations in all the storage solutions.

LDH increased on Day 3 to 3.4 × 10^−6^ units/mg protein and normalized on Day 7 to Day 1 levels (2.37 × 10^−6^ units/mg protein) in SSP+. LDH peaked on Day 3 (4.65 × 10^−6^ units/mg protein) and remained constant at 2 × 10^−6^ units/mg protein throughout storage in PAS-G. LDH increased on Day 5 (5.18 × 10^−6^ units/mg protein) and normalized on Day 7 to Day 3 levels (3.75 × 10^−6^ units/mg protein) in Tyrode's.

SSP+ exhibited a decline of 70 % (*p*-value <0.05) on Day 5 compared to Tyrode's.

### Platelet function markers

#### Aggregation

With collagen: Aggregation increased (*p*-value <0.05) on Day 7 in SSP+ with respect to Day 1 whereas, it was maintained throughout storage in PAS-G and Tyrode's.

Aggregation with collagen was similar between the storage solutions.

Without collagen: Aggregation without collagen was maintained throughout storage in SSP+ and PAS-G whereas, it increased (*p*-value <0.01) on Day 5 in Tyrode's with respect to Day 1.

Aggregation without collagen declined (p-value <0.05) in PAS-G on Day 5 compared to SSP+ and Tyrode's (Table 1).

**Table 1:** Function and oxidative stress in different storage solutions.

Storage period (days)	Platelet storage solutions (PAS)		Aggregation (405 nm)		ATP secretion (µg/ mL)	Superoxides (mM/mg protein)	Nitrites (mM/mg protein)
			With collagen	Without collagen			
1	SSP+		0.053 ± 0.01	0.039 ± 0.007	1812.65 ± 67.38	0.021 ± 0.005	1.93 ± 0.18
	PAS-G		0.046 ± 0.005	0.057 ± 0.007	1273.72 ± 127.0	0.072 ± 0.021	2.89 ± 0.183
	TB		0.056 ± 0.01	0.014 ± 0.006	1035.26 ± 50.96	0.033 ± 0.007	2.54 ± 0.07
3	SSP+		0.147 ± 0.008	0.1044 ± 0.02	549.33 ± 65.86*	0.09 ± 0.004	0.29 ± 0.032*
	PAS-G		0.09 ± 0.01	0.099 ± 0.02	220.74 ± 37.80*	0.08 ± 0.04	0.35 ± 0.024*
	TB		0.106 ± 0.02	0.092 ± 0.01	647.7 ± 78.0*	0.22 ± 0.03*	0.178 ± 0.05*
5	SSP+		0.125 ± 0.03	0.118 ± 0.02	269.04 ± 31.41*	0.03 ± 0.005	0.05 ± 0.01*#
	PAS-G		0.05 ± 0.01	0.031 ± 0.01	160.59 ± 58.25*	0.12 ± 0.014	0.06 ± 0.009*
	TB		0.122 ± 0.04	0.124 ± 0.04*	35.85 ± 6.76*	0.42 ± 0.06*#	1.03 ± 0.16*
7	SSP+		0.166 ± 0.03*	0.057 ± 0.006	235.44 ± 13.47*	0.27 ± 0.03*	0.45 ± 0.042*
	PAS-G		0.063 ± 0.02	0.051 ± 0.007	746.10 ± 92.61*	0.21 ± 0.025	0.99 ± 0.102*
	TB		0.15 ± 0.02	0.097 ± 0.01	202.46 ± 38.39*	0.133 ± 0.04	0.45 ± 0.14*

Values are means ± standard error (SE) of the platelets of five animals per group. Two-way ANOVA was applied between the groups (storage days) and sub groups (storage solutions) followed by Bonferroni's *post hoc* test using Graph Pad Prism-8 software. *P*-values <0.05 were considered significant.

Aggregation without collagen decreased in PAS-G compared to SSP+ and Tyrode's buffer on Day 5 (*p*-value <0.05). SSP+ exhibited increments in ATP secretion on Days 1 (*p*-value <0.0001) and 3 (*p*-value <0.05) whereas, it increased in PAS-G compared to other storage solutions on Day 7 (*p*-value <0.0001).

Superoxides decreased in PAS-G and SSP+ compared to Tyrode's buffer on Day 5 (*p*-value <0.0001). Nitrites decreased in SSP+ on Days 1 (*p*-value <0.05) and 5 (*p*-value <0.0001) and in PAS-G on Day 7 (*p*-value <0.05) compared to other storage solutions.

⁎ Represents significant changes between the groups with respect to Day 1.

# Represents significant changes between the groups with respect to Day 3.

### ATP secretion

ATP secretion declined (*p*-value <0.0001) with storage with respect to Day 1 in all the solutions.

ATP secretion decreased in Tyrode's and PAS-G compared to SSP+ on Day 1 (*p*-value <0.0001). ATP secretion was lower (*p*-value <0.05) in PAS-G on Day 3 and was higher on Day 7 (*p*-value <0.0001) with respect to SSP+ and Tyrode's (Table 1).

### Oxidative stress markers

#### Superoxides

Superoxides increased on Day 7 in SSP+ with respect to Day 1 (*p*-value <0.0001) whereas, they were similar throughout storage in PAS-G. Superoxides increased on Days 3 (*p*-value <0.01) and 5 (*p*-value <0.0001) in Tyrode's with respect to Day 1.

Superoxides dropped in SSP+ and PAS-G on Day 5 (*p*-value <0.0001) compared to Tyrode's (Table 1).

#### Nitrites

Nitrites dropped (*p*-value <0.0001) with storage with respect to Day 1 in all the solutions.

Nitrites increased in Tyrode's (*p*-value <0.05) and PAS-G (*p*-value <0.0001) compared to SSP+ on Day 1. Nitrites were lower (*p*-value <0.0001) in PAS-G on Day 5 and higher (*p*-value <0.05) on Day 7 compared to SSP+ and Tyrode's (Table 1).

### Lipid peroxidation

#### Conjugated dienes

Conjugated dienes peaked (*p*-value <0.0001) on Day 3 at 300 % (SSP+), 400 % (PAS-G) and normalized (*p*-value <0.0001) on Days 5 and 7 to their respective Day 1 levels. Conjugated dienes were maintained throughout the storage period in Tyrode's.

Conjugated dienes increased (*p*-value <0.0001) on Day 3 in PAS-G compared to SSP+ (68 %) and Tyrode's (2-fold) (Figure 1e).

#### Thiobarbituric acid reactive substances (TBARS)

TBARS decreased by 70 % (*p*-value <0.05) on Days 5 and 7 in PAS-G with respect to Day 1 (39 µM/mg protein) whereas, the levels were similar throughout storage in SSP+ (∼27 µM/mg protein) and Tyrode's (∼40 µM/mg protein).

TBARS were similar between the storage solutions on all storage days.

### Protein oxidation

#### Protein sulfhydryls (P-SH)

Sulfhydryls declined by 66 % and 85 % (*p*-value <0.0001) on Days 3 and 5 and normalized on Day 7 to Day 3 levels in SSP+. Sulfhydryls increased (*p*-value <0.01) by 4-fold in PAS-G and 10-fold in Tyrode's on Days 3 and 7.

SSP+ exhibited increments of 30-fold (*p*-value <0.0001) and 7-fold (*p*-value <0.0001) compared to Tyrode's and PAS-G, respectively on Day 1 (Figure 1c).

#### Advanced oxidation protein products (AOPP)

AOPP was maintained throughout the storage period in SSP+ and PAS-G whereas, it increased by 3-fold (*p*-value <0.0001) and 2-fold (*p*-value <0.01) on Days 3 and 5, respectively in Tyrode's compared to Day 1.

AOPP increased by 78 % (*p*-value <0.0001) on Days 3 and 5 in Tyrode's compared to PAS-G and SSP+ (Figure 1d).

### Antioxidant defenses

#### Superoxide dismutase (SOD)

SOD decreased by ∼85 % (*p*-value <0.01) on Day 3 and normalized to its respective Day 1 levels in all the storage solutions.

SOD increased by 5-fold (*p*-value <0.01) on Day 7 in SSP+ whereas, it increased by 7-fold (*p*-value <0.001) on Day 5 and ∼9-fold (*p*-value <0.0001) on Day 7 in PAS-G and Tyrode's, respectively in comparison to Day 3.

**Figure 2:**
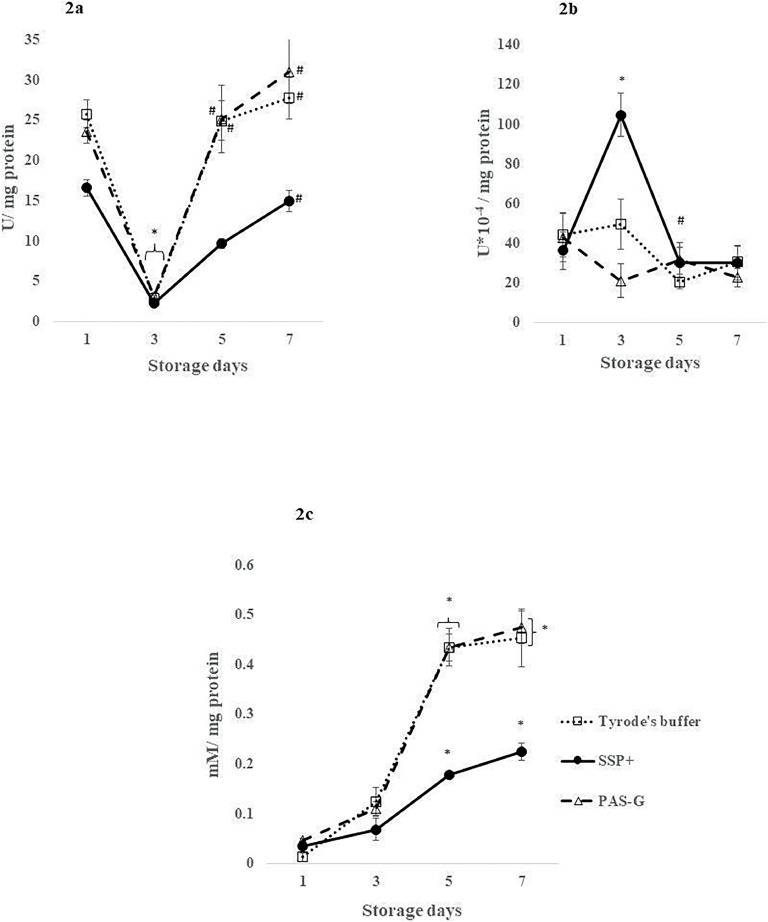
Antioxidant defenses in different storage solutions. Values are means ± standard error (SE) of the platelets of five animals per group. Two-way ANOVA was applied between the groups (storage days) and sub groups (storage solutions) followed by Bonferroni's *post hoc* test using Graph Pad Prism-8 software. *P*-values <0.05 were considered significant. *Represents significant changes between the groups with respect to Day 1. ^#^Represents significant changes between the groups with respect to Day 3. 2a. Superoxide dismutase (SOD): SOD increased in PAS-G and Tyrode's buffer compared to SSP+ on Days 5 and 7 (*p*-value <0.0001). 2b. Catalase (CAT): SSP+ exhibited increments in CAT activity compared to Tyrode's buffer (*p*-value <0.01) and PAS-G (*p*-value <0.0001) on Day 3. 2c. Glutathione: GSH levels increased in PAS-G and Tyrode's buffer compared to SSP+ on Days 5 and 7 (*p*-value <0.0001).

SSP+ showed decrements of 60 % (*p*-value <0.001) on Day 5 and ∼50 % on Day 7 compared to PAS-G (*p*-value <0.001) and Tyrode's (*p*-value <0.01) (Figure 2a).

#### Catalase (CAT)

CAT increased by 1-fold (*p*-value <0.001) on Day 3 and normalized (*p*-value <0.0001) on Day 5 to Day 1 levels in SSP+. CAT levels were maintained throughout storage in PAS-G and Tyrode's.

CAT declined by 80 % (*p*-value <0.0001) in PAS-G and 50 % (*p*-value <0.01) in Tyrode's on Day 3 compared to SSP+ (Figure 2b).

#### Glutathione (GSH)

Elevations were observed on Days 5 and 7 in all the storage solutions. Glutathione increased by 4-fold (*p*-value <0.05) and 5-fold (*p*-value <0.001) in SSP+, 8-fold (*p*-value <0.0001) and 9-fold (*p*-value <0.0001) in PAS-G and 30-fold (*p*-value <0.0001) in Tyrode's on Days 5 and 7, respectively compared to Day 1.

Glutathione increased by 1-fold (*p*-value <0.0001) on Days 5 and 7 in PAS-G and on Day 7 in Tyrode's compared to SSP+ (Figure 2c).

#### Total antioxidant capacity (TAC)

TAC showed similar variations in all the storage solutions.

TAC increased by 2-fold on Day 3 and 3-fold on Days 5 and 7 with respect to Day 1 (222 µM uric acid equivalents/L) in SSP+. TAC elevated by 100 % on Days 3 and 5 and 66 % on Day 7 with respect to Day 1 (322 µM uric acid equivalents/L) in PAS-G. TAC peaked by 100 % on Days 3 and 5, and 80 % on Day 7 with respect to Day 1 (402 µM uric acid equivalents/L) in Tyrode's.

### Platelet quality

#### Cell viability

The variations in cell viability were similar during storage with respect to Day 1 (0.405 ± 0.03) in SSP+. PAS-G exhibited decrements of 87 % (*p*-value <0.0001) on Day 7 compared to Day 1 (0.46 ± 0.02). Tyrode's also showed decrements by 82 % (*p*-value <0.0001) on Day 7 compared to Day 1 (0.53 ± 0.02).

Cell viability increased by 2-fold (*p*-value <0.01) and 3-fold (*p*-value <0.001) in SSP+ compared to Tyrode's and PAS-G, respectively towards the end of storage.

**Figure 3:**
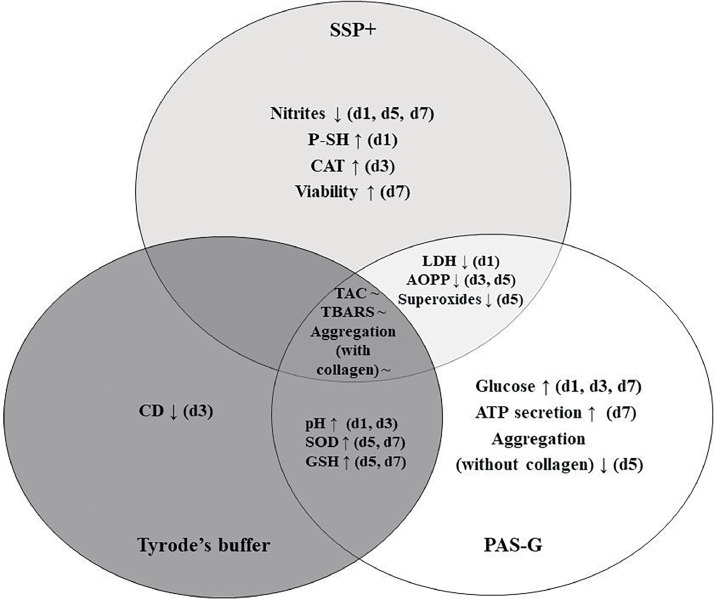
Comparison of storage solutions – SSP+, PAS-G and Tyrode's buffer. ATP: Adenosine triphosphate; AOPP: Advanced oxidation protein products; CAT: Catalase; CD: Conjugated dienes; GSH: Glutathione; LDH: Lactate dehydrogenase; P-SH: Protein sulfhydryls; SOD: Superoxide dismutase; TBARS: Thiobarbituric acid reactive substances; TAC: Total antioxidant capacity; d1, d3, d5, d7: storage Days 1, 3, 5, 7; ↑: significant increase compared to other storage solutions; ↓: significant decrease compared to other storage solutions; ∼ : maintained throughout storage.

The differential response of platelets in SSP+, PAS-G and Tyrode's has been summarized in Figure 3.

## Discussion

Platelets exhibited differential responses to three storage solutions in terms of platelet functions, metabolism, antioxidant defenses and OS. Platelet viability and antioxidant defenses improved in SSP+ whereas, antioxidant defenses such as superoxide dismutase (SOD) and glutathione (GSH) increased in PAS-G and Tyrode's buffer.

Antioxidant enzymes are activated due to high levels of reactive oxygen species (ROS). SOD dismutates superoxides to form H_2_O_2_ which is degraded by catalase (CAT) at higher concentrations and glutathione peroxidase (GPx) at lower concentrations.^23^ SOD levels are proportional to superoxide levels on Day 3 in all the storage solutions.

Dismutation of superoxides by SOD on Day 7 contributed to higher GSH levels on Days 5 and 7 in SSP+. ROS activated CAT which is reflected in increments in conjugated dienes on Day 3 and declines in sulfhydryls. Lower levels of H_2_O_2_ activated GPx on Day 5 leading to a decrease in CAT levels. Conjugated dienes further decreased on Days 5 and 7 due to the activation of SOD and CAT, and higher GSH levels. Protein oxidation was maintained throughout storage due to the stabilization effect of acetate on all the proteins in terms of AOPP.^24^ Sulfhydryls decreased as the plasma component of SSP+ could have served as a pool of ROS resulting in oxidation of thiols. Glucose consumption and lactate production are inversely proportional to pH during storage.^25^ Glucose peaked on Day 5 in SSP+ as the metabolic substrate switched from glucose (in plasma) to acetate and reversed on Day 7 evidenced by lower glucose levels. The pH increased on Days 5 and 7 due to the buffering effect of acetate. Activation of platelets in response to stimuli causes release of ATP and adenosine diphosphate (ADP) from the dense granules.^26^ ATP is converted to cyclic adenosine monophosphate (cAMP) by adenylate cyclase which is required for platelet responses. Lower levels of ATP facilitate activation and thereby aggregation.^27^ This was evident in the results of aggregation with collagen in SSP+. LDH is a marker of membrane stability and thereby, platelet viability.^28^ LDH and platelet viability were maintained throughout storage. However, platelet viability was significantly higher in SSP+ compared to PAS-G and Tyrode's. A number of coagulation factors and proteins are suspended in plasma^29^ which could account for the improved viability of platelets in SSP+.

PAS-G showed elevations in SOD whereas maintained CAT levels. This could be due to the scavenging activity of GPx which is reflected in the higher levels of GSH. PAS-G is beneficial in protecting lipids from oxidative damage as conjugated dienes and TBARS declined on Days 5 and 7. Acetate contributes to the stabilization of all proteins^24^ as evidenced in AOPP levels. Sulfhydryls increased throughout storage as PAS-G lacks plasma. Thus, PAS-G also prevented protein oxidation. PAS-G showed higher glucose consumption as evidenced by the decline in glucose levels till Day 5 indicating normal metabolic activity during storage. The pH was maintained due to the neutralizing effect of bicarbonate. Aggregation with collagen was maintained due to the decrease in the ATP levels. Lower ATP levels facilitate platelet responses and cause platelet aggregation.^27^ Hence, PAS-G aided in the mitigation of lipid peroxidation and protein oxidation, and preserved metabolism and function.

Superoxide levels were high on Day 5 and declined on Day 7 due to activation of SOD on Days 5 and 7 in Tyrode's. This is reflected in the redox status of platelets as evidenced by elevations in GSH and conserved CAT levels and lipid peroxidation products. Sulfhydryls increased throughout storage due to the activation of antioxidant defenses and absence of plasma. Thus, Tyrode's was beneficial in protecting thiol groups from oxidative damage. However, AOPP increased throughout storage as the susceptible proteins were prone to oxidation thereby forming dityrosine linkages. This could be due to the absence of the acetate component^24^ in Tyrode's. Glucose and pH were maintained throughout storage in Tyrode's due to the buffering effects of bicarbonate and dihydrogen phosphate. Decrements in ATP levels facilitate platelet responses leading to aggregation.^27^ Aggregation with collagen was maintained as ATP declined with storage. However, platelets aggregated without collagen on Day 5 indicating platelet response to OS conditions. Thus, Tyrode's could not preserve platelet function on Day 5.

To summarize, SSP+ is associated with platelet function, viability and antioxidant defenses (SOD, CAT and GSH); decreased primary lipid peroxidation products and maintained the susceptible protein groups in reduced state. Platelet function, antioxidant defenses such as SOD and GSH improved; lipids and thiols were protected from oxidation in PAS-G. SOD and GSH increased, and lipids and thiols were preserved in Tyrode's.

Data obtained in animal models do not necessarily translate into humans. Wistar rats were used to minimize the variations occurring in human blood samples. Each group of five Wistar rats were lab bred with similar genetics and environment. Thus, the outcome can be directly related to the interventions in the experimental design.

## Conclusion

SSP+ and PAS-G are more effective in maintaining platelet efficacy till Day 7 compared to Tyrode's buffer. SSP+ could maintain viability throughout storage. Thus, PAS-G and SSP+ were better than Tyrode's buffer in terms of platelet responses to OS during storage. These responses of platelets from Wistar rats can lay foundations for further studies in human samples.

## Funding

This research did not receive any specific grant from funding agencies in the public, commercial, or not-for-profit sectors.

## Ethics approval

Animal care and maintenance were in accordance with the ethical regulations (841/b/04/CPCSEA).

## CRediT authorship contribution statement

**Magdaline Christina Rajanand:** Writing – original draft, Formal analysis, Investigation. **Anusha Berikai Ananthakrishna:** Investigation, Writing – original draft. **Vani Rajashekaraiah:** Conceptualization, Writing – review & editing, Resources, Supervision.

## Conflicts of interest

The authors have no competing interests to declare that are relevant to the content of this article.
